# Transcending the brain disease versus disorder dichotomy: a critical realist perspective on psychiatric disorders

**DOI:** 10.1192/bjb.2025.8

**Published:** 2025-10

**Authors:** Mohammed Al Alawi, Abdullah Al Ghailani, Hamed Al Sinawi

**Affiliations:** 1 Department of Behavioral Medicine, College of Medicine and Health Sciences, Sultan Qaboos University, Muscat, Sultanate of Oman; 2 Oman Medical Specialty Board, Muscat, Sultanate of Oman; 3 Department of Behavioral Medicine, Sultan Qaboos University Hospital, University Medical City, Muscat, Sultanate of Oman

**Keywords:** Philosophy, history of psychiatry, phenomenology, psychosocial interventions, causal inference

## Abstract

In this opinion article, we discuss the application of critical realism as an alternative model to the biopsychosocial model in the understanding of psychiatric disorders. Critical realism presents a stratified view of reality and recognises mental disorders as emergent phenomena; that is, their full explanation cannot be reduced to explanations at any lower level of biological processes alone. It thus underscores the significance of the depth of ontology, the interaction between agency and structure, and the context dependency and complex nature of causality. Critical realism provides the conceptual and epistemological basis for a more subtle understanding of the aetiology of psychiatric conditions, which is polyfactorial and includes biological, psychological and social dimensions. Through the realisation of the conceptual and applicative shortcomings in the biopsychosocial model, critical realism promises to advance the understanding of mental disorders and enable a more holistic approach to the problem of people with mental disorders.

The debate over the question of whether psychiatric disorders should be viewed as ‘brain diseases’ has been long-standing and polarising. A disruption in neurobiology and brain circuitry, which suggests a brain disease model, is thereby forwarded as the reason for psychiatric conditions.^1^ Opponents argue that this biomedical reductionism underscores the critical psychological, social and experiential dimensions of mental illness.^2^ This is consistent with a more general debate regarding biological psychiatry’s relative emphasis on neurological pathology versus psychosocial perspectives emphasising broader determinants.

To explore this debate, we performed a literature search of a number of databases, including PubMed, PsycINFO, Google Scholar and JSTOR, using search terms such as ‘critical realism’, ‘biopsychosocial model’, ‘psychiatry’ and ‘causality’, targeting studies written in English from the mid-1970s to July 2024. Articles were selected based on the relevance of the theoretical discussion or empirical application of critical realism and the biopsychosocial model in psychiatry. Exclusion criteria included non-peer-reviewed work, opinion pieces that do not have scholarly references, and papers not from journals specifically about mental health. The major themes were identified from the philosophical and practical differences between articles applying critical realism and articles within the biopsychosocial model. These themes were then synthesised to highlight how critical realism provides a more significant explanation for psychiatric disorders and more holistic treatment models. This article presents the results of this synthesis.

## Critical realism and psychiatry

Critical realism differentiates between three distinct layers of reality: the empirical (what can be directly observed), the actual (events that occur, regardless of whether they are observable) and the real (the underlying mechanisms that generate these events).^3^ In the context of psychiatry, the ‘actual’ level refers to the underlying processes or events that contribute to psychiatric conditions, although these may not be immediately evident in clinical assessments.^4^ A clear example of critical realism’s framework can be applied to the understanding of major depressive disorder.

- Empirical level: at this stage, clinicians focus on the observable symptoms of depression, such as persistent low mood, lack of motivation, fatigue and disruptions in sleep or appetite. These symptoms are assessed through clinical interviews, patient self-reports and standardised diagnostic instruments like the Patient Health Questionnaire-9 (PHQ-9).^1^
- Actual level: moving beyond what can be observed, the actual level encompasses significant life events or processes that, while not directly visible, play a crucial role in the development of the disorder. For example, experiences such as childhood trauma, unresolved grief or prolonged psychosocial stress are actual events that contribute to the onset of the condition. Although these factors may not surface during a clinical assessment, they are essential to understanding the patient’s mental health.^4^
- Real level: at the most fundamental layer, the real level involves the underlying biological mechanisms that interact with actual events to produce the observable symptoms. In the case of depression, these mechanisms may include genetic predispositions, neurochemical imbalances or a dysregulated stress–response system. These deeper generative factors set the stage for actual events (such as trauma or stress) to influence the patient’s mental health.^5^

This layered approach enables clinicians to view psychiatric disorders as complex and multifaceted, necessitating treatment that targets not only the observable symptoms but also the underlying causes at both the actual and real levels.^6^

## Critical realism and causality in psychiatry

Importantly, critical realism rejects simple linear causality and views causation as implicating interactions across the stratified levels of reality mediated by the generative mechanisms.^7^ This epistemic model is in accord with a multifactorial biopsychosocial aetiology that is now believed to be responsible for most psychiatric disorders.^8^ The disruptions in brain circuitry often provide necessary but insufficient causal conditions for the emergence of disorders, with the psychological, social and environmental mechanisms as indispensable component causes.^9^

## Critical realism and explanatory pluralism

Critical realism also accepts what is known as explanatory pluralism, that different valid explanations from diverse perspectives can coexist.^10^ This permits the integration of insights not just from biological psychiatry, with its concern for neurotransmitters and circuits, but also from psychodynamic explorations of subjective experience or social psychiatry’s framing of the structural determinants. Such a move allows open, complex causation to be addressed within a rationality that respects the competing knowledge claims of different approaches.^11^

## Striking the balance

Critical realism allows psychiatry to develop multi-level explanations regarding deficits, which can range from neurological to mental/social causes, without resorting to reductionism.^12^ The ‘brain disease’ view can be incorporated, although balanced by other perspectives that include existential, cultural or politico-economic considerations. What results is a coherent, stratified, causally pluralistic model to understand psychopathology.^13^

## Critical realism and the biopsychosocial model

The biopsychosocial model played a crucial role in moving away from purely biomedical reductionism, but it faces several conceptual and practical challenges in its approach to understanding mental disorders.^6,12,14^ Although it integrates biological, psychological and social factors, the model often treats them as isolated, static components. This compartmentalisation leads to a fragmented clinical approach, where the complex interactions between these factors are often overlooked. In contrast, critical realism offers a layered ontological framework that not only acknowledges these factors but also highlights their dynamic interconnections across different levels of reality.^5^

The biopsychosocial model, while aiming to integrate various domains, often gravitates towards biological determinism in psychiatry, where medication tends to be prioritised over psychosocial interventions. Pilgrim critiques the biopsychosocial model for its insufficient consideration of the social structures and power dynamics that significantly influence mental health outcomes.^14^ In contrast, critical realism acknowledges that social inequalities, systemic structures and historical power imbalances are integral to the causal processes, shaping both actual life events (such as trauma or unemployment) and the observable symptoms (like anxiety or depression) in clinical settings.^6,15,16^ Sims-Schouten & Riley expand on this by introducing critical realist discourse analysis as a method to explore how these structures and factors interact to shape experiences of mental health, offering a nuanced approach to understanding people’s mental health problems within a broader societal and institutional context.^17^

Critical realism brings attention to the more profound generative mechanisms that essentially underpin psychiatric disorders, such as sociopolitical structures, economic inequality and historical trauma, in contrast to the biopsychosocial model, which favours focusing on immediate causes (such as individual stress or neurotransmitter imbalances).

The medicalisation of mental illness, particularly depression, has been criticised for focusing too heavily on biological causes while overlooking social and psychological influences. Bentall refers to this as the ‘medicalisation of misery’, arguing that reducing depression to a biological disorder ignores the important role of life experiences and social context.^18^ This aligns with critical realism, which emphasises that mental health problems arise from a complex interaction between biological, social and structural factors.^18^ Recent critiques^19–21^ further stress that global mental health approaches often miss these deeper sociopolitical realities, advocating for a more holistic perspective that addresses both individual and social dimensions of mental illness.

These real-world mechanisms influence the circumstances surrounding real-life occurrences (such as social isolation or childhood trauma) that lead to the emergence of mental illness.^6^ Rather than acknowledging these social factors as essential determinants of mental health outcomes, the biopsychosocial model, on the other hand, frequently sees them as contextual or incidental.

Although the biopsychosocial model provides a valuable framework for integrating biological, psychological and social factors, its application in practice can vary significantly. Some practitioners may adopt a more mechanistic approach that risks oversimplifying complex psychiatric presentations, whereas others may employ a nuanced, multifactorial perspective. A critical realist approach can complement the biopsychosocial model by offering an additional lens to explore interactions across multiple levels of causality – from neurobiological processes to sociocultural structures – thereby enriching clinical understanding.^5,15,22^

This approach may enable a deeper understanding of the causal mechanisms behind mental disorders, beyond the immediate biopsychosocial interactions, encompassing the generative mechanisms that give rise to these interactions.^16,23^

## Critical realism: agency and structure

Critical realism focuses on the dialectical relationship between agency and structure. Although the biopsychosocial model acknowledges some importance of individual and social factors, critical realism further delves into how societies’ structures and cultural systems enable and, at the same time, restrict individuals’ abilities to act. This approach allows for a more dynamic understanding of how social determinants of mental health operate and change over time.^22,24^ In other words, it provides insights into how personal agency and social structures interact in the development and treatment of mental disorders. The critical realism approach attests to the casual complexity of mental disorders and argues against monocausal explanations while at the same time explaining context-dependent causality. It thus sharply contrasts with some applications of the biopsychosocial model since they may tend implicitly towards an additive causation model. Critical realism, owing to the concerns with contingent and context-sensitive notions of causal relations, propels a more flexible and contextually sensitive take on making sense of and addressing mental disorders.^25^

## Philosophical rigour

Critical realism offers a thoughtful philosophical perspective for exploring foundational assumptions about knowledge, reality and causality. Although the biopsychosocial model remains a vital framework for understanding mental illness by integrating biological, psychological and social dimensions, critical realism can complement this approach by encouraging a deeper reflection on the connections between these dimensions.^25,26^ By addressing challenges such as reductionism and dualism, it opens possibilities for broadening our understanding of the complex factors shaping psychiatric phenomena. At the same time, the practical application of critical realism’s principles remains a challenge. Nevertheless, its focus on ontological depth, causal complexity and epistemological pluralism has the potential to support psychiatry’s ongoing efforts to develop a more integrated and multidimensional understanding of mental illness.

## Critical realism and falsifiability

A more nuanced understanding of falsifiability is offered by critical realism, particularly in the context of psychiatric research. Whereas conventional falsifiability functions at the empirical level, allowing for the testing and measurement of observable symptoms and results, critical realism recognises that deeper generative mechanisms at the actual and real levels are more difficult to falsify.^5^ Randomised controlled trials (RCTs) are a useful tool for evaluating clinical interventions for psychiatric disorders at the empirical level. These interventions may include medications or psychotherapies. RCTs provide a clear framework for falsifiability because observable outcomes such as symptom reduction can support or refute the effectiveness of the treatment.

However, falsifiability becomes more complex at the real and actual levels. The actual level includes life events or psychological pressures that may influence the emergence of a disorder but are not immediately observable. Similarly, the real level involves underlying biological mechanisms, such as genetic predispositions or neurochemical imbalances, which are difficult to directly falsify.^27^ Instead, these mechanisms are investigated through indirect evidence, such as patterns found in case studies or longitudinal research. For example, while it may not be possible to directly refute the role of trauma in depression, consistent patterns across cases suggest that trauma plays a significant role.^14^

Thus, critical realism allows for a layered approach to falsifiability, where deeper mechanisms are understood indirectly through their effects on observable phenomena, whereas empirical observations can be tested directly. This approach acknowledges the complexity of mental illnesses and the multiple factors that contribute to their development, helping to distinguish fact from opinion.

## Conclusions

Although the biopsychosocial model has done much to further our understanding of mental disorders, critical realism provides a sophisticated enough philosophical framework that helps to apply ontological depth to the complex interplay between agency and structure and cause, as well as philosophical rigour. Such an approach broadens contributions to our understanding of mental illness and suggests pathways for the research, diagnosis and treatment that should be sensitive to the complex realities of the disorder in mind.

## Data Availability

The data that support the findings of this article are available from the corresponding author on reasonable request.

## About the authors

**Mohammed Al Alawi** is Assistant Professor and Consultant of Liaison Psychiatry at Sultan Qaboos University, Muscat, Sultanate of Oman. **Abdullah Al Ghailani** is a senior psychiatry resident at Sultan Qaboos University Hospital, University Medical City, Muscat, Sultanate of Oman. **Hamed Al Sinawi** is Department Head and Senior Consultant of old age psychiatry at Oman Medical Specialty Board, Muscat, Sultanate of Oman.
